# A Distended Bladder Mistaken for a Possible Ovarian Tumor

**Published:** 1884-09-20

**Authors:** C. Henri Leonard


### A DISTENDED BLADDER MISTAKEN FOR A POSSIBLE OVARIAN TUMOR.

By C. Henri Leonard, M.D.
Case reported to Wayne County Medical Society.

While at the first thought the heading just read seems preposterous, in that such a case should happen, yet I am free to say that, to one only superficially acquainted with the history of pelvic and abdominal enlargements and their various symptoms, the error is one quite easy to make, if the catheter be not used as an instrument of diagnosis. Indeed, I can imagine cases that, without this final method of eliminating a bladder trouble, would puzzle one quite well versed in gynæcological manipulation.

A fibro-cyst of the uterus might also be mistaken for an enlarged bladder, due to inordinate and long-continued distension. The case that I have to report was thought, at times, by the attendant, to possibly be this variety of abdominal growth.

The history of the case, in brief, is as follows: Mrs. X., aged about thirty-two, has been confined to her bed some six or eight weeks with quite a prominent abdominal enlargement, this enlargement continually increasing, though slow of growth. Before taking to her bed there was constipation of the bowels, some trouble with the bladder, though she seemed to herself to be passing the requisite amount of urine. Some six or eight weeks prior to my seeing her, she had what was, and as I still think, an attack of remittent fever. Coupled with this was a steady, though not very intense, pain in the lower and central abdominal region. This called attention to this point for examination by the regular attendant and the patient herself. A tumor was seen, though not of very great size; this was painful on pressure, and did not seem to have any connection with the uterus, so far as external abdominal palpation could determine. As the patient was confined to her bed, and with little or no appetite, she gradually became more and more emaciated, and this brought the "tumor" more and more prominently out to the notice of the patient, husband and medical attendant. She was passing what was considered the normal quantity of water every day, and so neither the attendant nor friends thought of the enlargement as being due to a distended bladder. As time wore on the pain became excessive and opiates were required; this still farther kept the bowels inactive, and so increased still more, by the filling of the rectum back of the bladder with fecal matter and thus pressing the bladder forward, the enlargement noticed in the lower portion of the abdomen. With this excess of pain came *dribbling of the urine* at times, due, as they thought, to the pressure of the tumor upon the bladder. The symptom of "dribbling urine," I would wish to impress upon you, is, in ninety cases out of every hundred, due to an over-distended bladder. The bladder is paralyzed and cannot contract to empty itself completely, yet may partially do it, as in this case. Only the day before I saw her she passed as much urine as she was wont to do in health, as she supposed—though, evidently, she did not; and yet here was this dribbling of water all the night and so much of the day as had passed before I saw her.

As I came into the room she lay on her back with her knees drawn up as far as she could get them towards the abdomen; face was contracted, pain intense over the lower abdominal region whether pressure was made over it or not. This suggested peritonitis to the attendant, and, indeed, the whole look of the patient was that of peritonitis. Her friends had thought she must die, and she had given up all hope herself.

Palpation showed a sharply defined tumor, slightly to the left of the median line—probably pushed there by the sigmoid flexure of the colon. The history, as I got it, led me to announce that the growth had been too rapid for a true cyst of the ovary. I asked if she had passed her water regularly every day, for palpation seemed to show the tumor to be the bladder rather than a fibro-cyst of the uterus—though I have seen them take on as rapid enlargement as had this "tumor." The husband and patient both insisted that it could not be that, as she had passed her water every day; farther, had just passed some just before I came in, and that it had been "dribbling away for a day or so." This was just the point, I told them, that confirmed my impression; the urine had dribbled away because *the bladder was over-distended.*

Pulling out my pocket-case, I adjusted my catheter and told them I would make my diagnosis good in a few moments. The patient did not relish the experiment, for she was "sure I must be wrong." However, the catheter was introduced, when the urine started to flow in a full stream. A teacup was handed me to let it run in. I asked for a larger vessel, when a bowl was handed me. I then said, bring me a pail. By this time they all saw the truth of my diagnosis, and for full ten minutes the water kept running, pressure being gently applied above the pubes to assist the paralyzed viscus to empty itself. Relief from the intense pain came before we had got half through with the hidden fountain that we had tapped.

The full amount taken was beyond what I ever dreamed the human bladder could be made to hold, for it was one large chamber full and a full half of another.

The patient went into a healthy sleep after we had adjusted a compress, and made up for that lost previously. The organ needed to be emptied for several days with the catheter, but the patient made a speedy and perfect recovery on general tonic treatment.

In a recent number of the New York Medical Journal is the following account of an "Over Distension of the Bladder Mistaken for an Ovarian Cyst." It was reported by Dr. T. A. Emmet:

Dr. T. A. Emmet had received a letter one Sunday afternoon asking him to meet the writer as soon as possible in consultation regarding a case of ovarian tumor, which had existed some six or eight weeks. Dr. Emmet remarked, on receiving the letter, that he could not imagine how an ovarian tumor could develop to a marked size in so short a time. The following Wednesday afternoon was fixed for the consultation, and when he was about to enter the room a young physician from the neighborhood met him, having been called in hastily while the woman was in convulsions. He said that, as the patient had not passed much water during the day, he hastily put together the two sections of a silver catheter which he had in his pocket, not having the third section with him, and began to draw off the urine. About a quart having escaped, he turned to empty the vessel, when the catheter was sucked from between his fingers up into the bladder. Dr. Emmet found the woman in deep coma. The abdomen was enormously distended, as much so as in any case of ovarian tumor which he had ever seen. The walls were so tense that he could scarcely obtain fluctuation, although it was evident that the distension was due to fluid. He sent out for an elastic catheter, with which he emptied the bladder, whereupon the tumor disappeared. An enormous amount of urine was withdrawn. After the bladder had been emptied, the silver catheter was felt projecting against the abdominal walls as through an ordinary sac. He had little difficulty in removing it with the forceps, unobserved. It seemed that before Dr. Emmet saw the patient she had been about the house, and there had been some vesical irritation, supposed to be due to the presence of an ovarian tumor. She was about thirty-four years of age. This was the second case that he had seen in which the catheter had been sucked up into the over-distended bladder during catheterization, and he asked if there was any explanation of a tendency to this accident in this condition. The case was interesting, also, from a diagnostic point of view. The patient had been going to the closet oftener than usual for nearly two months, passing a little water each time, but there was constantly developing a greater degree of vesical distension, which had been mistaken for an ovarian tumor by one who, under ordinary circumstances, certainly would not make a mistake regarding a tumor of the ovary. Dr. Emmet thought that, as a specialist, he himself might have been misled as to the true condition. He was told that the patient finally died in an apoplectic seizure, the result of renal disease.

Dr. Post said that he had once been called to make a post mortem examination in the case of a female child, four or five years of age, supposed by the attending physician to have been suffering with incontinence of urine. He found the bladder enormously distended with urine, and extending as high up as the umbilicus.—*Med. Age.*

